# Chronic Alcoholism and HHV-6 Infection Synergistically Promote Neuroinflammatory Microglial Phenotypes in the *Substantia Nigra* of the Adult Human Brain

**DOI:** 10.3390/biomedicines9091216

**Published:** 2021-09-14

**Authors:** Nityanand Jain, Marks Smirnovs, Samanta Strojeva, Modra Murovska, Sandra Skuja

**Affiliations:** 1 Joint Laboratory of Electron Microscopy, Institute of Anatomy and Anthropology, Rīga Stradiņš University, LV-1010 Riga, Latvia; marks.smirnovs@rsu.lv; 2 Institute of Microbiology and Virology, Rīga Stradiņš University, LV-1067 Riga, Latvia; samanta.strojeva@rsu.lv (S.S.); modra.murovska@rsu.lv (M.M.)

**Keywords:** microglia, neuroinflammation, chronic alcoholism, HHV-6, *Substantia Nigra*

## Abstract

Both chronic alcoholism and human herpesvirus-6 (HHV-6) infection have been identified as promoters of neuroinflammation and known to cause movement-related disorders. *Substantia Nigra* (SN), the dopaminergic neuron-rich region of the basal ganglia, is involved in regulating motor function and the reward system. Hence, we hypothesize the presence of possible synergism between alcoholism and HHV-6 infection in the SN region and report a comprehensive quantification and characterization of microglial functions and morphology in postmortem brain tissue from 44 healthy, age-matched alcoholics and chronic alcoholics. A decrease in the perivascular CD68+ microglia in alcoholics was noted in both the gray and white matter. Additionally, the CD68+/Iba1− microglial subpopulation was found to be the dominant type in the controls. Conversely, in alcoholics, dystrophic changes in microglia were seen with a significant increase in Iba1 expression and perivascular to diffuse migration. An increase in CD11b expression was noted in alcoholics, with the Iba1+/CD11b− subtype promoting inflammation. All the controls were found to be negative for HHV-6 whilst the alcoholics demonstrated HHV-6 positivity in both gray and white matter. Amongst HHV-6 positive alcoholics, all the above-mentioned changes were found to be heightened when compared with HHV-6 negative alcoholics, thereby highlighting the compounding relationship between alcoholism and HHV-6 infection that promotes microglia-mediated neuroinflammation.

## 1. Introduction

A linear double-stranded DNA virus, human herpesvirus-6 (HHV-6) is a ubiquitous β-herpesvirus, first isolated from the peripheral blood mononuclear cells of patients with lymphoproliferative disorders [1,2]. It has been postulated that the salivary glands act as the major and persistent reservoir of the virus in humans, especially given its frequent detection in saliva and the suggested role of saliva in viral transmission [3,4,5,6]. A small proportion (1–2%) of the cases has been reported to be associated with vertical transmission of the virus during pregnancy [7,8]. A vast majority of children by the age of 2 years become seropositive as the seroconversion begins as soon as the protection from passive maternal antibodies starts to wear off [1]. The virus is known to exist in two close variants: HHV-6A and HHV-6B. Whilst HHV-6A has not yet been etiologically linked to any disease [1,9,10], HHV-6B is known to cause Roseola infantum (sixth’s disease; exanthema subitum), which occasionally presents with neurological complications like febrile seizures or encephalitis [9].

The neurotropic nature of the virus, coupled with its ability to infect a broad range of cells/tissues in vivo including the endothelium [11] and brain [12,13], leads to a wide spectrum of clinical complications. Additionally, it has been speculated that it plays a role in central nervous system (CNS) -related diseases including meningoencephalitis [14,15], Alzheimer’s disease [16,17], multiple sclerosis [18,19], and mood disorders [20,21]. Often, active infection in immunocompromised patients has been described as a fulminant multifocal demyelinating disease [22,23,24]. Although active HHV-6 infection predominately affects the limbic system [25], other brain regions like the olfactory cortex have also been reported to be altered [26,27,28]. Reports of patients presenting chorea-like involuntary movements and other movement disorders in HHV-6 infection [25,29,30] leads us to speculate the possible involvement of basal ganglion, a region associated with the “motor circuit” and related movement disorders [31].

*Substantia Nigra* (SN), also known as the black substance, is the dopaminergic neuron-rich region that modulates motor movement and reward functions as part of the basal ganglia [32]. It is divided anatomically into two subregions: *Pars Compacta* (SNpc) and *Pars Reticulata* (SNpr). SNpc is composed of densely packed neurons with high concentrations of neuromelanin (a dark polymer pigment formed by the oxidative polymerization of dopamine or noradrenaline) [32,33], while SNpr mainly consists of GABAergic (gamma-aminobutyric acid) inhibitory neurons. SNpc exerts its effects on the motor cortex either via direct or indirect pathways. In the direct pathway, projections from SNpc reach D1 (dopamine) receptors in the striatum, causing inhibition of the GPi (globus pallidus interna) and SNpr, leading to disinhibition of the thalamic nuclei and allowing the required movement to occur [33]. In the indirect pathway, projection synapses with D2 striatal receptors cause the GPe (globus pallidus externa) to be relatively excited, thereby inhibiting subthalamic nuclei and activating GPi and SNpr, which finally leads to inhibition of motor function [34].

Alcohol (ethanol) has long been an established modifier of brain activity. At low-to-moderate doses, it has been recognized as a dose-dependent stimulant of motor activity, while at high doses, it has been shown to cause ataxic effects (uncontrolled, abnormal movements) [35,36,37]. Further, the development of alcohol tolerance and addiction represent another modulating effect of alcohol on the dopamine-mediated reward system of the brain. Chronic alcohol use dysregulates the immune system, causing increased susceptibility to infections and inflammatory reactions in the brain and peripheral organs [38]. Studies in animal models have shown that β-carbolines and their derivatives (found in abundance in alcoholic beverages), upon in vivo metabolization, form compounds resembling 1-methyl-4-phenylpyridinium ions (MPP^+^), a neurotoxicant involved in the pathogenesis of idiopathic Parkinson’s disease [39]. Moreover, these derivatives have been shown to induce early-onset neurodegenerative changes, glial activation in SNpc, and a significant long-term decrease in spontaneous motor activity [39].

Both HHV-6 and alcohol have been identified as promoters of neuroinflammation. Neuroinflammation, a broad term that does not correlate to the classical characteristics seen in peripheral inflammation, represents a chronic, CNS-specific, and glial-mediated inflammation-like response [40]. Microglial cells, the immune sentinels in the brain parenchyma that are thought to orchestrate a potent neuroinflammatory response [41], account for about 5–10% of the total cell population in the brain. Together with nonparenchymal brain macrophages (perivascular, meningeal, and choroid plexus macrophages), each of these cell types occupies a specific niche, thereby covering the entire CNS [42]. Under physiological conditions, mature microglial cells (so-called “resting/surveillance microglia”) are usually highly ramified with fine, long processes and small somata; however, upon activation in response to pathogens or DAMPs (damage-associated molecular patterns) [42], a change in surface molecule expression along with transformation from ramified to ameboid (so-called “activated microglia”) shape via a multistep activation cascade is seen [43,44]. While the initial microglial response may provide neuroprotection by eliminating the distress source and restoring tissue homeostasis, in cases of persistent stimuli like HHV-6, microglial cells can become chronically active, resulting in upregulation of pro-inflammatory cytokines which jeopardizes neuronal survival [45,46,47].

Evidence from previous studies led us to investigate the potential role and synergistic effects of these partners-in-crime, i.e., chronic alcoholism and HHV-6 infection, in the disruption of brain homeostasis and increased neuroinflammatory response in the SN region of the brain. Additionally, we explored the various subpopulations of microglial cells, providing valuable insights into the stages of transformation which these cells undergo, their function, and quantification in both normal and alcoholism-related conditions, thereby advancing our knowledge of brain homeostasis in the hope of achieving better diagnostic and treatment modalities for patients.

## 2. Materials and Methods

### 2.1. Autopsy Brain Tissue Collection and Characteristics

Brain autopsy specimens from 44 individuals were provided by the Latvian State Centre for Forensic Medical Examination. Postmortems were performed between 7 and 30 h after death. A complete medical history including evidence of alcoholism (confirmed by ethanol levels in the blood coupled with tissue changes seen in the liver, pancreas, and heart) was provided by a certified pathologist. The autopsy brain samples were collected between 2007–2012 and preserved in paraffin blocks following the appropriate protocols. Brain tissues for the SN region were obtained using the human brain map atlas [48]. Patients with infections, diabetes, or respiratory system pathologies were excluded from the study. The inclusion criteria included previous history of alcoholism in accordance with the criteria established by Harper et al. [49].

The autopsies of alcoholic subjects evidenced alcohol abuse and liver cirrhosis. Additionally, ascites, pancreatitis, and cardiomyopathy were found [50]. In the laboratory, patient specimens were grouped based on age and exposure to alcohol, and assigned internal codes as shown in Table 1. Group A included 13 control individuals with a median age of 31 ± 6.79 years and no history of alcohol consumption. Control group individuals presented no neuropathological abnormalities upon postmortem examination and had no history of major psychiatric illness [50]. Group B included 13 age-matched alcoholic individuals with a median age of 31 ± 4.85 years. Group C consisted of 18 non-age-matched alcoholic individuals with a median age of 49.5 ± 8.66 years.

The protocol for the present study was approved by the Ethics Committee of Rīga Stradiņš University (Decision No. 6-1/12/9) dated 26 November 2020, as per the provisions of the Declaration of Helsinki. Informed consent was provided by the next of kin for the autopsy.

### 2.2. Immunohistochemistry (IHC), Double Immunohistochemistry, and Immunofluorescence (IF)

Routine hematoxylin and eosin (H&E) staining was done using formalin-fixed paraffin-embedded (FFPE) tissue sections which were examined using a light microscope to verify that the slides contained the regions of interest for the present study (SNpc and SNpr). Upon verification, the sections were prepared for standard immunohistochemistry (IHC) and immunofluorescence (IF) reactions [51]. Following the manufacturer’s guidelines, tissue sections were incubated overnight with primary antibodies, as described in Table 2.

CD68 (Cluster of differentiation 68), also known as LAMP-4 (Lysosomal associated membrane protein 4), is a transmembrane glycoprotein that is associated with the cellular, endosomal, and lysosomal compartments [52,53]. A scavenger receptor that binds oxidized low-density lipoproteins (oxLDL), CD68 is highly expressed in cells of macrophage lineage with low expression in lymphocytes, fibroblasts, and endothelial cells [54]. Whilst CD68 can be expressed by resting microglia, it is commonly considered as a marker of activated microglia due to its role in phagocytotic activities [55,56].

CD11b (Cluster of differentiation 11b), a commonly used peptide marker for activated and resting microglia, is the α-subunit of CR3 (Complement receptor 3), an integrin that is involved in adhesion processes [57,58]. Iba1 (Ionized calcium-binding adapter molecule1) is a peptide encoded by the gene AIF1 (Allograft inflammatory factor 1). As a cytoplasmic protein, Iba-1 is primarily and constitutively expressed by both activated and resting microglial cells in the brain tissue. Like CD68, it has also been implicated in phagocytic processes [59,60].

HHV-6 (20) is an antibody that is raised against viral lysate and can be used to detect the HHV-6A and HHV-6B subtypes [61,62]. The presence of the respective antigens was determined either by using the HiDef DetectionTM HRP Polymer system (Cell Marque, Rocklin, CA, USA) and 3,3′ diaminobenzidine (DAB) tetrahydrochloride kit (DAB + Chromogen and DAB + Substrate buffer, Cell Marque, Rocklin, CA, USA) or the goat antimouse IgG (H + L) antibody, Alexa Fluor^®^ 488 conjugate (Thermo Fisher Scientific, Invitrogen, UK, 1:300). For nuclear visualization, counterstaining with Mayer’s hematoxylin (Sigma Aldrich, St. Louis, MO, USA) or 4′,6-diamidino-2-phenylindole (DAPI) (Thermo Fisher Scientific, Invitrogen, UK, 1:3000) was done, respectively.

In order to detect the two different antigens (double IHC staining), both the HiDef DetectionTM HRP Polymer system and the HiDef DetectionTM Alk Phos Polymer system were used successively, followed by counterstaining with a DAB substrate kit (brown color) and Permanent Red Chromogen Kit (red color), respectively (Cell Marque, Rocklin, CA, USA). Tissue rinsing, dehydration, clearing, and mounting in Roti^®^ Histokitt (Carl Roth, Karlsruhe, Germany) or Prolong Gold with DAPI (Thermo Fisher Scientific, Invitrogen, UK) was done as per the manufacturer’s protocols. Treatment with Sudan Black B solution (Sigma Aldrich, St. Louis, MO, USA) was done to reduce tissue autofluorescence. Positive and negative controls were prepared for each antibody reaction (as per the manufacturer’s guidelines). Negative controls were prepared using tris(hydroxymethyl)amino -methane (TRIS) solution in place of the primary antibody.

After conventional immunostaining, the expression of marker proteins was manually evaluated using a Leica light microscope (LEICA, LEITZ DMRB, Wetzlar, Germany) and Glissando Slide Scanner (Objective Imaging Ltd., Cambridge, UK) in 10 randomly selected visual fields per sample per region. IHC markers were considered positive if brown-stained cell or cell clusters were observed. A quantitative scoring system was used for positively stained cells or cell groups by two independent observers. Immunofluorescence (IF) was used to confirm the localization of the viral proteins using a Nikon Eclipse Ti-E confocal microscope (Nikon, Brighton, MI, USA).

### 2.3. DNA Extraction and HHV-6 Detection Using Nested Polymerase Chain Reaction (nPCR)

Fine tissue sections of 0.5–1 mm^2^ were obtained from the formalin-fixed, paraffin-embedded (FFPE) brain tissue blocks to isolate DNA using the commercial blackPREP FFPE DNA kit (Analytik Jena AG, Germany) following the manufacturer’s protocol. The concentration of the extracted DNA was determined using a NanoDrop ND-1000 Spectrophotometer (Thermo Fisher Scientific, Waltham, MA, USA). The measurements were taken according to the manufacturer’s protocol. The quality of genomic DNA (gDNA) was determined by detecting the β-globin gene sequence with the polymerase chain reaction (PCR) method using appropriate primers [63]. DNA obtained from *Substantia Nigra* (SN) tissue was considered as qualitative if 200 bp products were acquired by PCR. We found that all samples were β-globin positive.

Detection of viral genomic sequences in the isolated DNA from SN tissue was done using nested polymerase chain reaction (nPCR). HHV-6 was detected by a two-step PCR reaction with primers targeting the viral U3 gene that encodes the main capsid proteins for both variants HHV-6A and HHV-6B [64]. Each experiment was performed along with positive, negative, and water controls. For positive controls, HHV-6A and HHV-6B genomic DNA (Advanced Biotechnologies Inc., Columbia, MD, USA) was used. For negative controls, DNA samples obtained from practically healthy HHV-6 negative blood donors were used.

### 2.4. HHV-6A and HHV-6B Variant Detection Using HindIII Restriction Endonuclease

HHV-6A and HHV-6B were differentiated according to the methodology described by Lyall and Cubie [65]. A two-step PCR reaction was performed again to differentiate between the two subtypes of the virus with primers targeting the HHV-6 large tegument protein (LTP) gene following HindIII restriction analysis. The primers used for the PCR reaction are summarized in Table 3. The obtained nPCR amplicons were digested with HindIII restriction endonuclease (Thermo Scientific, Waltham, MA, USA) according to the manufacture’s protocol. HindIII cleaves the HHV-6B positive sample into two fragments of 66 bp and 97 bp (base pair), but does not cleave HHV-6A at all. All PCR results were visualized using 1.7% agarose electrophoreses gel.

### 2.5. Viral Load Determination Using Real-Time PCR (RT-PCR)

Positive DNA samples from FFPE whole SN region (SNpc and SNpr) blocks were used for HHV-6 load detection using the HHV-6 Real-TM Quant (Sacace Biotechnologies, Como, Italy) commercial kit in accordance with the manufacturer’s instructions. The β-globin gene was used as the internal control which serves as an amplification control for each processed specimen and can aid in the identification of possible reaction inhibitions. HHV-6 viral load > 10 copies/10^6^ cells were considered to be significantly increased.

### 2.6. Statistical Analysis

The data collected from each visual field (quantitative counting of the immune-positive structures in IHC) were stored in spreadsheets using MS Excel (Microsoft Office 365) Distribution of the dataset was checked using the Shapiro-Wilk test for normality (*p* < 0.05 indicates a violation of normality). Since all our datasets violated the conditions of normality, nonparametric tests were used for analysis. Kruskal-Wallis ANOVA (analysis of variance) was used for intergroup analysis with post hoc tests and Bonferroni correction. The intragroup analysis was done using the related-samples Wilcoxon signed rank test. *p* < 0.05 was considered statistically significant. The statistical analysis was done using SPSS (IBM Corp. Released 2020; IBM SPSS Statistics for Windows, Version 27.0; Armonk, NY, USA: IBM Corp), while graphs were prepared using R studio and MS Excel.

## 3. Results

### 3.1. Alcoholics Showed a Significant Decrease in CD68+ Cells Than Controls in Both Gray and White Matter

White matter showed more CD68 positive (CD68+; significant only in SNpr) cells in *Substantia Nigra* (Table 4), in line with previous studies which indicated that the basal level of phagocytosis was higher in white matter than gray matter [66,67,68]. Overall, a decrease in the number of CD68+ cells in both gray and white matter was noted in alcoholics when compared with controls (Table 4). Although the decrease was not significant in SNpc, a significant decrease in CD68+ cells was seen in the gray matter of SNpr between Group A and Group C (*p* = 0.025). Similarly, in the white matter of SNpr, a significant decrease was seen between controls and alcoholics (Groups A–C *p* = 0.000; Groups B–C *p* = 0.021).

**Figure 1:**
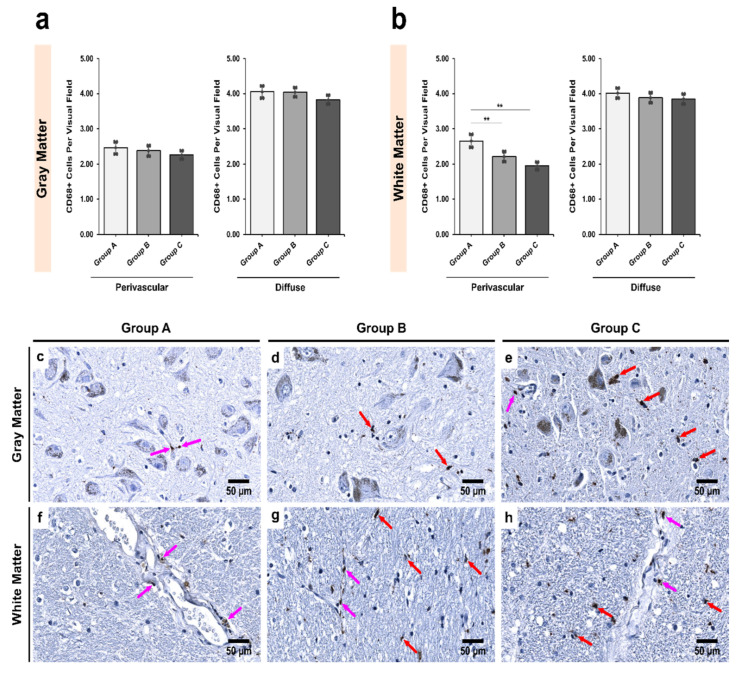
Intergroup analysis of CD68+ cells per visual field in *Pars Compacta* (SNpc) for the three studied groups in both (**a**) gray and (**b**) white matter. The analysis was done for both perivascular and diffuse locations. The bar plots indicate the average number of CD68+ cells ± S.E. (Standard Error) seen per visual field. ** indicates a significant difference between the groups (*p* < 0.05 with Bonferroni correction is considered significant). Distribution of CD68+ cells per visual field in (**c**,**f**) Group A (controls); (**d**,**g**) Group B (age-matched alcoholics) and (**e**,**h**) Group C (non-age-matched alcoholics). Pink arrows indicate perivascular CD68+ cells while red arrows indicate diffuse CD68+ cells. Original magnification, 400×. Scale bars, 50 µm.

In *Pars Compacta* (SNpc), alcoholics showed less diffuse CD68+ cells than controls; however, the difference was not found to be statistically significant in both gray and white matter (Figure 1; Kruskal Wallis *p* = 0.784 and 0.683, respectively). Perivascularly, however, there were significant differences observed in the white matter (Figure 1b). Alcoholics showed significantly less CD68+ cells than controls (Groups A–B and A–C; *p* = 0.003 and 0.027, respectively). No significant differences were noted in the gray matter perivascularly (*p* = 0.374).

**Figure 2:**
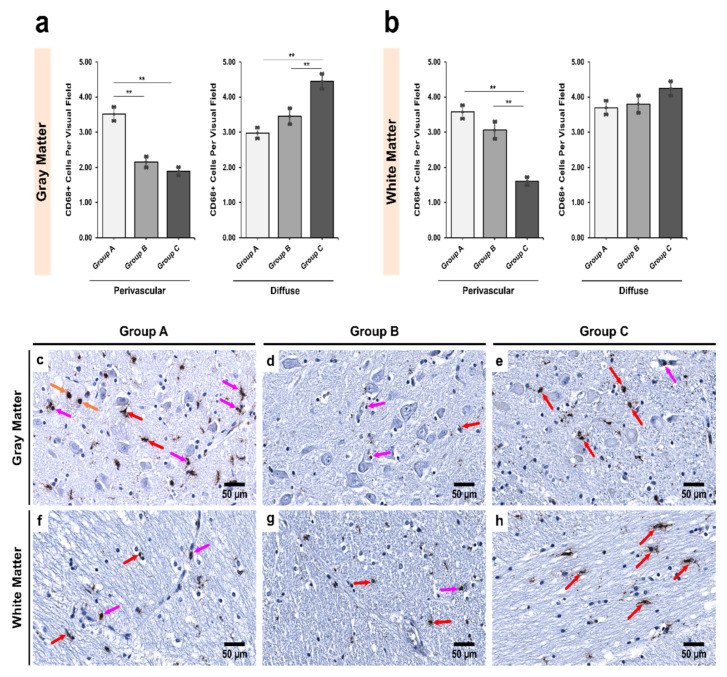
Intergroup analysis of CD68+ cells per visual field in *Pars Reticulata* (SNpr) for the three studied groups in both (**a**) gray and (**b**) white matter. The analysis was done for both perivascular and diffuse locations. The bar plots indicate the average number of CD68+ cells ± S.E. (Standard Error) seen per visual field. ** indicates a significant difference between the groups (*p* < 0.05 with Bonferroni correction is considered significant). Distribution of CD68+ cells per visual field in (**c**,**f**) Group A (controls); (**d**,**g**) Group B (age-matched alcoholics) and (**e**,**h**) Group C (non-age-matched alcoholics). Pink arrows indicate perivascular CD68+ cells; red arrows indicate diffuse CD68+ cells while orange arrows indicate monocytes (seen in the lumen of micro-vessel). Original magnification, 400×. Scale bars, 50 µm.

In *Pars Reticulata* (SNpr), perivascularly, both alcoholic groups (Groups B and C) showed a decrease in the number of CD68+ cells when compared with controls (Group A) in both gray and white matter (Figure 2). A significant decrease in nonmatched alcoholics was noted in both gray and white matter when compared with controls (*p* < 0.001). Further, a significant decrease was noted in the gray matter between age-matched alcoholics and controls (*p* < 0.001). Conversely, alcoholics showed an increase in the number of diffuse CD68+ cells when compared with controls in both the gray and white matter. While significant differences were observed in the gray matter (Groups A–C *p* < 0.001; Groups B–C *p* = 0.024), the differences were not statistically significant in the white matter (*p* = 0.187).

### 3.2. Alcoholics Showed a Significant Increase in Iba1+ Cells Compared to Controls in Both Gray and White Matter

The distribution of Iba1+ cells was found to be significantly more abundant in the white matter in the SNpc (Groups A and B) and SNpr (Group A; Table 5) when compared with gray matter. In contrast to the average number of CD68+ cells, alcoholics showed significantly more Iba1+ cells when compared with controls (Table 5) in both SNpc and SNpr. In both gray and white matter of SNpc, controls had significantly less Iba1+ cells in comparison to both age-matched alcoholics (*p* < 0.001) and non-age-matched alcoholics (*p* < 0.001). In SNpr, however, there were no significant differences between controls and age-matched alcoholics. Rather, significant differences in both gray and white matter were noted between Groups A–C and Groups B–C (*p* < 0.001).

**Figure 3:**
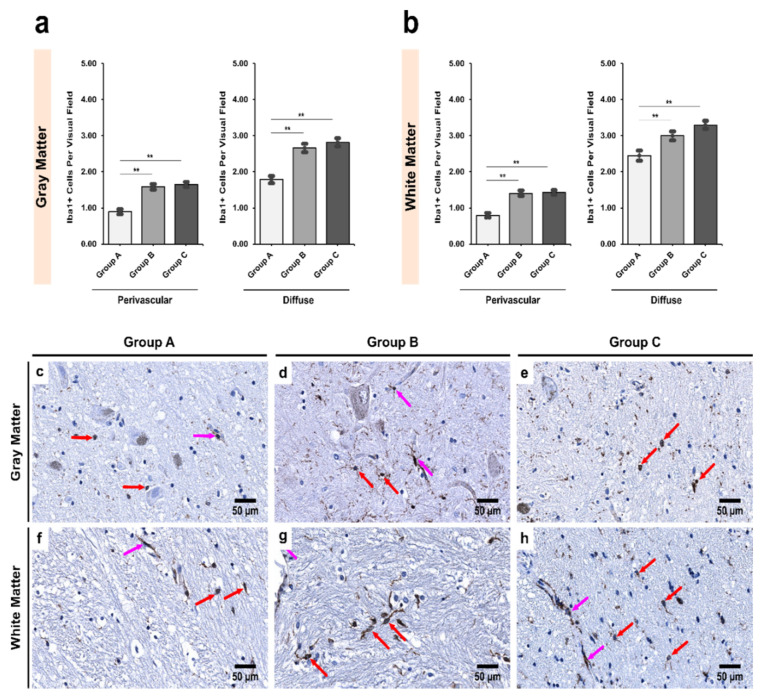
Intergroup analysis of Iba1+ cells per visual field in *Pars Compacta* (SNpc) for the three studied groups in both (**a**) gray and (**b**) white matter. The analysis was done for both perivascular and diffuse locations. The bar plots indicate the average number of Iba1+ cells ± S.E. (Standard Error) seen per visual field. ** indicates a significant difference between the groups (*p* < 0.05 with Bonferroni correction is considered significant). Distribution of Iba1+ cells per visual field in (**c**,**f**) Group A (controls); (**d**,**g**) Group B (age-matched alcoholics) and (**e**,**h**) Group C (non-age-matched alcoholics). Pink arrows indicate perivascular Iba1+ cells while red arrows indicate diffuse Iba1+ cells. Original magnification, 400×. Scale bars, 50 µm.

In the SNpc, both age-matched and chronic alcoholics showed a significant increase in perivascular Iba1+ cells in both the gray and white matter compared with the controls (*p* < 0.001; Figure 3). Similar results were observed in the diffuse locations in both gray and white matter (*p* < 0.001; Figure 3). The effect of age-related changes could be ignored here due to significant differences between the controls and age-matched alcoholics (*p* = 0.001—perivascular and *p* = 0.001—diffuse).

**Figure 4:**
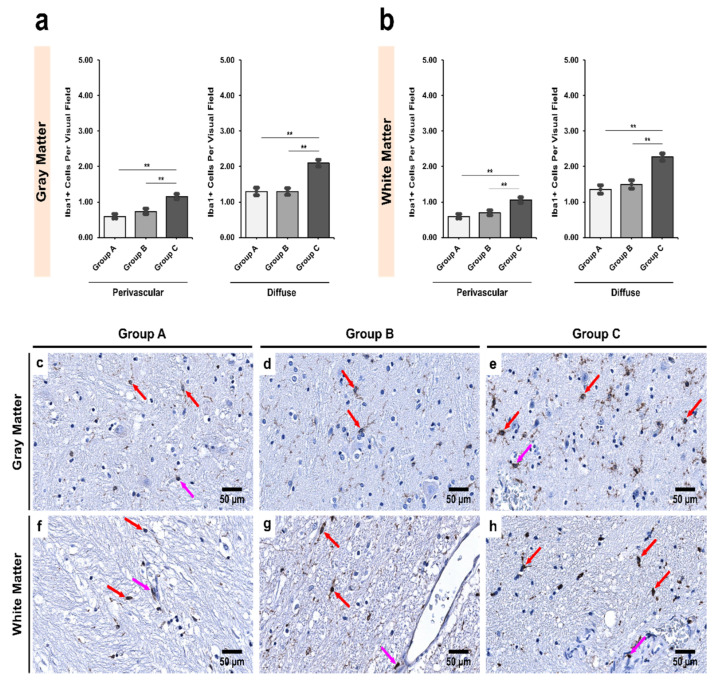
Intergroup analysis of Iba1+ cells per visual field in *Pars Reticulata* (SNpr) for the three studied groups in both (**a**) gray and (**b**) white matter. The analysis was done for both perivascular and diffuse locations. The bar plots indicate the average number of Iba1+ cells ± S.E. (Standard Error) seen per visual field. ** indicates a significant difference between the groups (*p* < 0.05 with Bonferroni correction is considered significant). Distribution of Iba1+ cells per visual field in (**c**,**f**) Group A (controls); (**d**,**g**) Group B (age-matched alcoholics) and (**e**,**h**) Group C (non-age-matched alcoholics). Pink arrows indicate perivascular Iba1+ cells while red arrows indicate diffuse Iba1+ cells. Original magnification, 400×. Scale bars, 50 µm.

In the SNpr, however, no statistically significant differences were observed between controls and age-matched alcoholics (Figure 4), though age-matched alcoholics had more Iba1+ cells (Table 5). Furthermore, the chronic alcoholics showed a significant increase in Iba1+ cells compared to controls in both gray and white matter (*p* < 0.001), and in both perivascular (*p* = 0.006) and diffuse locations (*p* = 0.009). Similar results were obtained upon comparison of age-matched and chronic alcoholics (*p* = 0.002 and 0.001, respectively; Figure 4).

### 3.3. Alcoholics Showed Significantly More CD11b+ Cells Than Controls in Both Gray and White Matter

In SNpc, there was a significant increase in CD11b+ population in non-age-matched alcoholics compared with controls (Table 6). There were also significant differences between age-matched alcoholics and controls in both gray and white matter (*p* = 0.000 and 0.034, respectively). In SNpr, although there were more CD11b+ cells noted in alcoholics in both gray and white matter, the difference was not statistically significant. Like Iba1 and CD68, CD11b was also found to be more abundant in white matter than gray matter.

**Figure 5:**
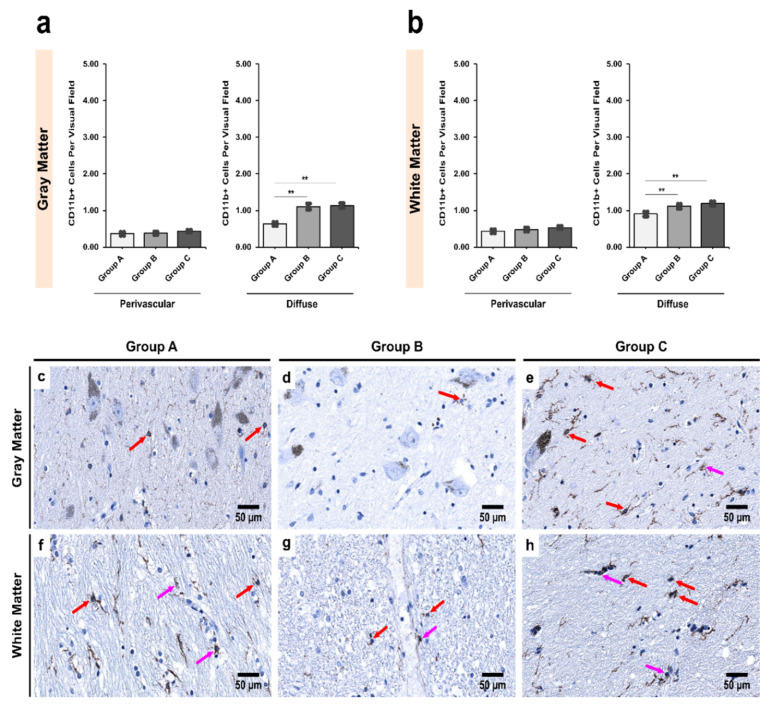
Intergroup analysis of CD11b+ cells per visual field in *Pars Compacta* (SNpc) for the three studied groups in both (**a**) gray and (**b**) white matter. The analysis was done for both perivascular and diffuse locations. The bar plots indicate the average number of CD11b+ cells ± S.E. (Standard Error) seen per visual field. ** indicates a significant difference between the groups (*p* < 0.05 with Bonferroni correction is considered significant). Distribution of CD11b+ cells per visual field in (**c**,**f**) Group A (controls); (**d**,**g**) Group B (age-matched alcoholics) and (**e**,**h**) Group C (non-age-matched alcoholics). Pink arrows indicate perivascular CD11b+ cells while red arrows indicate diffuse CD11b+ cells. Original magnification, 400×. Scale bars, 50 µm.

In the SNpc, the alcoholics showed a mild increase in CD11b+ cells perivascularly in both the gray and white matter, though the results were not statistically significant (*p* = 0.330 and 0.368, respectively). However, in the diffuse locations in both the gray and white matter, there was a significant increase between controls and age-matched alcoholics (*p* = 0.001 and 0.009, respectively; Figure 5). A similar significant increase was noted between controls and chronic alcoholics in both the gray and white matter (*p* = 0.002 and 0.048, respectively; Figure 5).

**Figure 6:**
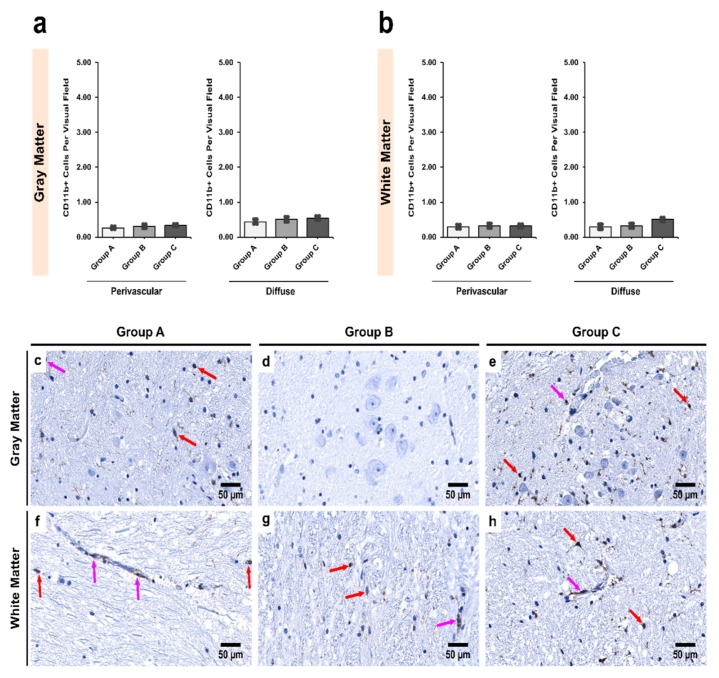
Intergroup analysis of CD11b+ cells per visual field in *Pars Reticulata* (SNpr) for the three studied groups in both (**a**) gray and (**b**) white matter. The analysis was done for both perivascular and diffuse locations. The bar plots indicate the average number of CD11b+ cells ± S.E. (Standard Error) seen per visual field. Distribution of CD11b+ cells per visual field in (**c**,**f**) Group A (controls); (**d**,**g**) Group B (age-matched alcoholics) and (**e**,**h**) Group C (non-age-matched alcoholics). Pink arrows indicate perivascular CD11b+ cells while red arrows indicate diffuse CD11b+ cells. Original magnification, 400×. Scale bars, 50 µm.

In the SNpr, however, no significant differences in CD11b+ cell numbers were noted in either gray or white matter in both perivascular (*p* = 0.218 and 0.614, respectively) and diffuse (*p* = 0.528 and 0.811, respectively) locations (Figure 6).

### 3.4. Exposure to Alcohol Induces Stronger Iba1 Expression Than CD11b in Microglial Cells

**Figure 7:**
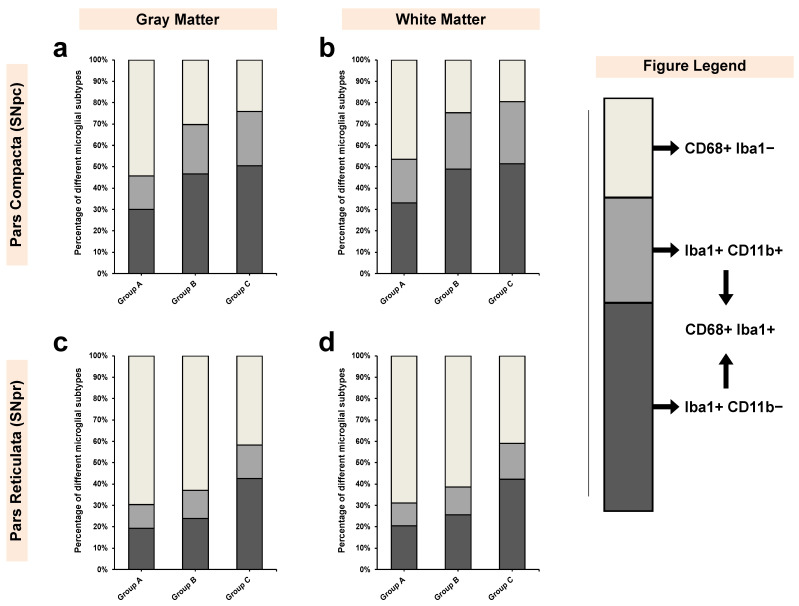
Distribution of different microglial subpopulations (in %) in both SNpc and SNpc for all three groups. The data has been shown for (**a**,**c**) gray and (**b**,**d**) white matter. The figure legend describes the various subpopulations identified including CD68+/Iba1− and CD68+/Iba1+. Within CD68+/Iba1+, two subtypes were identified namely, Iba1+/Cd11b+ and Iba1+/CD11b−.

Overall, we observed four distinct microglial subpopulations based on the antibodies used in the present study (CD68, Iba1, and CD11b). These subpopulations were CD68+/Iba1+; CD68+/Iba1−; Iba1+/CD11b+ and Iba1+/CD11b− (Figure 7). In controls, the vast majority of microglial cells expressed CD68 but did not express Iba1 (CD68+/Iba1−) in either gray or white matter in SNpc and SNpr. However, a sharp decrease of this subpopulation was noted in age-matched alcoholics, which was followed by continued decline in non-age-matched alcoholics. Conversely, prolonged exposure to alcohol led to an increase in expression of Iba1+ microglial subpopulation (an increase by factor of about 1.7× to 2.4×).

**Figure 8:**
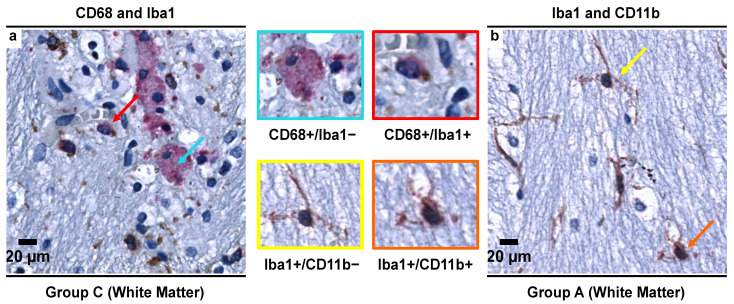
Representative images from double immunohistochemical staining (using two IHC markers in a single slide) showing different microglial subpopulations in the SN region. (**a**) Double CD68/Iba1 IHC staining showing two distinct subpopulations of microglia—CD68+/Iba1− (blue arrow) and CD68+/Iba1+ (red arrow) in the white matter of the alcoholics (Group C); (**b**) Double Iba1/CD11b IHC staining showing two distinct subpopulations of microglia—Iba1+/CD11b− (yellow arrow) and Iba1+/CD11b+ (orange arrow) in the white matter of the controls (Group A). In both images, brown color (DAB) indicates Iba1+ structures while red color (Permanent red) indicates CD68+ or CD11b+ structures. Original magnification, 400×. Scale bars, 20 µm.

Within the Iba1+ microglial population, both cells with and without CD11b expression increased with prolonged exposure to alcohol (non-age-matched alcoholics). In both the regions of *Substantia Nigra* (SNpc and SNpr), we found that there were discrepancies in the distribution of CD11b+ and CD11b− subpopulations. In all regions, CD11b− microglia was the dominant subpopulation (even in controls; Figure 8). In fact, while the increase of CD11b+ subpopulation in alcoholics was around 1.4× to 1.6× in comparison with controls, the increase of CD11b− subpopulation was around 1.6× to 2.2× compared with controls.

### 3.5. Morphological Characterization of Microglial Subpopulations

**Figure 9:**
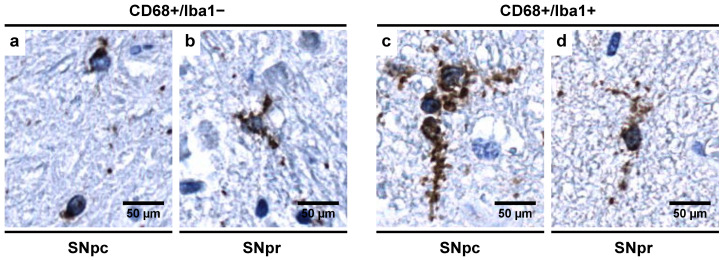
Morphology of different microglial subpopulations as seen in (**a**,**c**) SNpc and (**b**,**d**) SNpr. In CD68+/Iba1− subpopulation (dominant in controls in SN region), (**a**) microglia is sparsely branched in SNpc while (**b**) show complexity in cell processes in SNpr. This subpopulation represents microglia in “*controlled phagocytotic activated state*”. In CD68+/Iba1+ subpopulation (dominant in alcoholics in SN region), (**c**,**d**) microglia shows dystrophic changes seen as by appearance of beaded processes in SNpc and SNpr. Original magnification, 400×. Scale bars, 50 µm.

The findings from the previous section suggest a rather sequential and preferential expression of certain immunohistochemical markers as microglial cells undergo the different stages of morphological transformations in response to prolonged alcohol exposure. The CD68+/Iba1− subpopulation, which was dominant in the controls (Figure 9a,b), showed different morphologic characteristics in SNpc and SNpr. Whilst in SNpc, microglia were sparsely branched, in the SNpr, microglia showed complexity in cell processes. On the other hand, in alcoholics, the CD68+/Iba1+ subpopulation was dominant, which was less ramified and possessed fewer branches and/or beaded processes (Figure 9c,d). Furthermore, such dystrophic changes were more prominently noticed in the SNpr.

### 3.6. Alcoholics Showed Detectable HHV-6 Positivity and Viral Load

**Figure 10:**
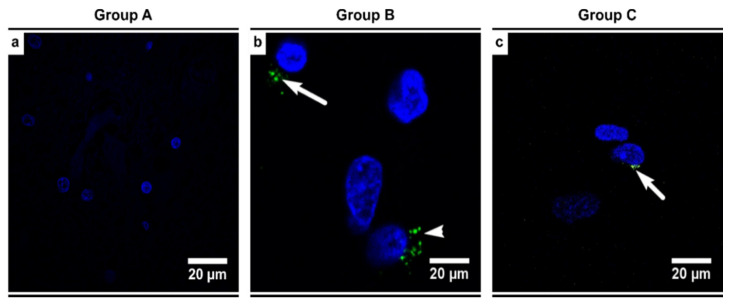
Representative images illustrating HHV-6 viral proteins (seen as green spots) using immunofluorescence (IF) in the gray matter of the alcoholics (confocal microscopy; original magnification × 1000). White arrows indicate the clusters of HHV-6 antigens—(**a**) controls (Group A) showed no positivity whilst (**b**) age-matched alcoholics (Group B) and (**c**) chronic alcoholics (Group C) showed HHV-6 immunopositivity in the cell cytoplasm.

As expected, none of the individuals in the control group (Group A) showed positive HHV-6 immunostaining (Figure 10a). This was also confirmed with a negative result for HHV-6 genomic sequences (using nPCR). Amongst the alcoholics (Groups B and C), 25% (8/31) of the individuals were found to be positive for HHV-6 genomic sequences, with viral detection seen more commonly in the gray matter (88% of positive individuals) than the white matter (63% of positive individuals) in the SN region (Figure 10b,c). In all positive individuals, only the HHV-6B variant was detected, with an average viral load of 101,207.97 copies/10^6^ cells.

### 3.7. Alcohol and HHV-6 Infection in a Synergistic and Potentiating Relationship Cause Disruption of Homeostasis in the SN Region

**Figure 11:**
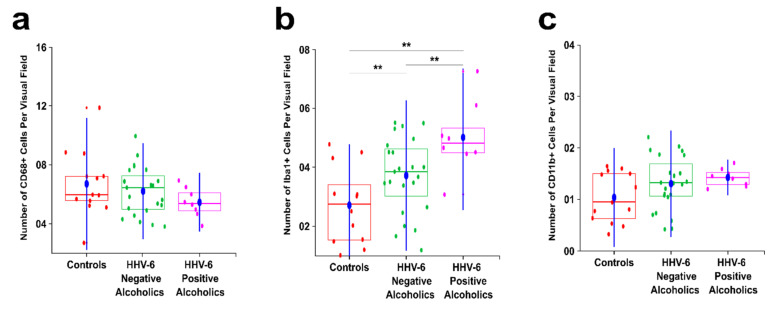
Intergroup analysis between HHV-6 positive alcoholics, HHV-6 negative alcoholics, and controls. The blue dot indicates the mean of the respective group. Red color indicates controls, green color indicates HHV-6 negative alcoholics and pink color indicates HHV-6 positive alcoholics. ** indicates a significant difference between the groups (Kruskal Wallis ANOVA *p* < 0.05 with Bonferroni correction is considered significant). (**a**) Boxplot showing the average number of CD68+ cells per visual field in the SN region; (**b**) Boxplot showing the average number of Iba1+ cells per visual field in the SN region; (**c**) Boxplot showing the average number of CD11b+ cells per visual field in the SN region.

To evaluate the synergistic relationship between alcoholism and HHV-6, we next evaluated their combined effects on microglia (CD68, CD11b and Iba1). This was achieved by comparing the HHV-6 positive alcoholics (from both Groups B and C) to the HHV-6 negative alcoholics (from both Groups B and C) and the controls (Figure 11). HHV-6 positive alcoholics showed greater decrease in the number of CD68+ cells per visual field than HHV-6 negative alcoholics, though the difference was not statistically significant (*p* = 0.287).

For Iba1+ cells, there was a significant increase in HHV-6 positive alcoholics from both controls (2× increase; *p* = 0.002) and HHV-6 negative alcoholics (1.34× increase; *p* = 0.028). Further, although the difference between CD11b+ cells remained nonsignificant (*p* = 0.265), HHV-6 positive alcoholics showed an increase of about 1.4× from controls and 1.1 × from HHV-6 negative alcoholics (Figure 11c). This discrepancy in the expression induction of Iba1+ and CD11b+ in microglial cells demonstrates the synergistic effects of HHV-6 infection in terms of potentiating a stronger alcoholic induction of Iba1 and a relatively milder induction of CD11b (Figure 7), leading to the emergence of Iba1+/CD11b− subpopulation (Figure 8).

## 4. Discussion

The potential for alcoholism and viral infection to disrupt brain homeostasis has long been a topic of interest and research. Yet, a lot of questions remain unanswered. In the present study, we report a comprehensive characterization of microglial functions, morphology, and quantification in individuals in the SN region, along with the specific changes that microglial cells undergo due to co-exposure to alcohol and HHV-6 infection. Since neuroinflammatory changes are also seen in the normal aging process, the inclusion of age-matched alcoholics (Group B) gives us the power to specifically exclude aging as an underlying factor in the study. Additionally, with the inclusion of postmortem human brain tissue from relatively young adults, the insights gained reflect the closest possible representation to changes in the adult human brain.

### 4.1. Abundance of CD68+/Iba1− Microglial Subpopulation in Controls Indicates a Special Physiological “Controlled Phagocytotic Activated State”

Iba1 has been reported to be a universal marker for all microglial subpopulations [56,69,70]. However, emerging evidence suggests otherwise [71]. Recent experiments on the murine microglia BV2 cell line showed that silencing Iba1 protein in microglial cells leads to a significant decrease in cell migration, proliferation, and cell adhesion capabilities [72]. Further, the authors demonstrated that such silencing leads to an increase in phagocytic activity along with upregulation of P2 × 7 (ATP-activated P2 purinergic receptors) functioning [72]. This is of immense interest since, in our study, we found that in controls, 45–55% of the entire microglial population in SNpc and 65–70% of the entire microglial population in SNpr did not demonstrate Iba1 expression (Figure 7 and Figure 8). Such findings indicate that in the SN region, microglial cells show reduced motility but a high level of phagocytic activity. This specific state of activated microglia is different from the one induced by disease-specific activating mechanisms [72].

The above-mentioned findings compelled us to further explore the reasons behind the prevalence of such specific microglial phenotype in controls. Firstly, the SN region has one of the highest densities of microglial cells (around 12% of the total brain microglia population), with SNpr being denser than SNpc (Table 4) [73,74]. Secondly, we speculate that such a high proportion of microglia in a relatively smaller region (compared with cortex) gives microglial cells the ability to monitor the entire region sufficiently without the need for excessive migration-related activities. Thirdly, in a recent study by Ayata et al., the authors demonstrated that the microglial clearance activity rate is different in different regions of the brain, with microglial epigenetic regulators restricting clearance activity in certain regions (striatum, cortex) while promoting it in others (cerebellum) [75]. The authors further linked this epigenetic regulation to the rate of neuronal attrition [75].

Finally, De Biase et al. found that in a functional state (i.e., in the absence of pathology), microglia in SNpr exhibited structural complexity in the cell processes with a significant percentage of cell volume being occupied by lysosomes (10% of the cell volume), while in SNpc, microglial cells were sparsely branched with 6% cell volume being occupied by lysosomes [73], in line with our results (Figure 9a,b). In both SNpc and SNpr, the authors found that the lysosomal content (by % volume occupied) in microglia is more than that of other regions of the basal ganglia [73], leading us to correlate this with the increased baseline phagocytotic activity due to non-expression of Iba1 [72]. Our results, combined with those presented in the literature, lead us to postulate that the microglia in the SN region, under normal physiological conditions, are in what we are calling a “*controlled phagocytic activated state*” which needs further investigation (especially in the context of Parkinson’s and other neurodegenerative diseases).

### 4.2. Decrease in the Number of CD68+ Microglia with Increase in Iba1+ Expression Shows Microglial Dystrophy Which Leads to Compensatory Mobility from Perivascular to Diffuse Locations

Alcohol has been shown in vitro (BV2 microglial cell line) to accelerate the production of reactive oxygen species (ROS) in microglial cells, which subsequently leads to activation of PARP (poly(ADP-ribose) polymerase) and oxidative-stress sensitive TRPM2 (transient receptor potential melastatin-related 2) channels, ultimately causing microglial death [76]. Additionally, alcohol exposure in rats leads to an increase in dystrophic microglia [77]. Although the classical hypothesis posits that hyperactivated and hypertrophic microglia are responsible for responding to stress or injury and are responsible for neurodegenerative changes, emerging evidence from animal studies suggests that alcohol-induced suppression of normal microglial function (dystrophic changes) also leads to neuronal cell death and subsequent neurodegenerative changes [77,78,79].

Long-term and constant microglial activation is thought to cause immune exhaustion and microglial burn-out, leading to dystrophic changes which are seen as cells being less ramified and possessing fewer branches and/or beaded microglial processes (Figure 9c,d), which ultimately causes cytorrhexis (accidental cell death) [80,81]. Additionally, previous studies have indicated that both microglial dystrophy and microglial activation are simultaneous processes [82]. Such dystrophic changes alter the distribution and quantity of CD68+ microglial cells in alcoholics (Table 4), which then require compensatory changes.

We hypothesize two major compensatory changes that the microglial cells demonstrate. The first is an increase in motility-related activities. This is supported by the hypothesis that if silencing of Iba1 leads to decreased cellular motility and migration [72], the increased expression would have the opposite effects. Moreover, it has been shown that such migrations are needed to remove cellular debris and provide support to salvageable cells in regions of damage [41,77,83]. It appears that in the SN region, such migratory movements are rather directed towards diffuse locations, away from perivascular locations (Figure 1 and Figure 2). The second change is the decrease in phagocytic activities. Our results also support this notion, as we saw a decrease in the number of CD68+ cells (since CD68 is related to phagocytosis; Table 4) and the fact that Iba1 expression probably leads to a slowdown in phagocytosis [72]. It has been demonstrated that alcohol may be cytotoxic to microglia since there is a complete lack of phagocytic microglia after an acute alcohol binge [78]. Although we saw an increase in Iba1 expression, the authors reported otherwise [77,78]. We speculate that such differences are due to the region of the brain investigated, which further bolsters the findings that microglial regulation is not uniform across the different regions of the brain [73,75,84].

### 4.3. Alcohol and HHV-6 Infection Co-Induce and Accelerate Microglial Dystrophy

In a study by Bortolotti et al., spheroid 3D models of peripheral blood monocyte-derived microglia from healthy donors were infected with HHV-6A [45]. The authors reported a significant uptick in Iba1 and substance P (associated with neuroinflammation) expression and concluded that microglial cells were permissive to HHV-6A infection, which ultimately resulted in increased Aβ1-42 (beta-amyloid; implicated in Alzheimer’s disease) expression [45]. Further, they demonstrated that HHV-6A infection leads to microglial cell migration to the site of infection (under paracrine effect), bringing their results in-line with our findings. However, in contrast to our findings, the authors reported that the ramified microglial morphology was predominant in response to HHV-6A infection [45]. Such morphological differences can be attributed to the effects of alcohol, as discussed above. Like HHV-6A, its cousin HHV-6B has also been shown to infect and dysregulate microglial function [85,86].

Leibovitch et al. demonstrated increased Iba1 expression in HHV-6B infected marmosets (primates) suffering from experimental autoimmune encephalomyelitis [87]. According to the fertile field hypothesis, in a heightened immune state (which can be induced by pathogens or self-antigens), there is a lower threshold for autoreactivity due to the expansion of autoreactive cells against the backdrop of immune response disbalance [88]. Whilst based on multiple clinical studies, the role of alcohol and HHV-6 individually remains controversial in the pathogenesis of Alzheimer’s disease (AD) and multiple sclerosis (MS), we hypothesize that joint exposure to alcohol and HHV-6 is the key trigger required for clinical manifestations and progression of AD and MS (this hypothesis requires in vitro and in vivo validation). In such a case, we hypothesize, that HHV-6A and HHV-6B exposure (or reactivation, immune suppression, alcohol-mediated activation) creates a fertile field which would probably lead to AD- and MS-like symptoms, while alcohol exploits the lowered autoreactivity threshold to potentiate the neuroinflammatory and neurodegenerative changes.

### 4.4. Dominance of Iba1+/CD11b− Microglial Subpopulation in Alcoholics Leads to Chronic Inflammation, Hyperalgesia, and Allodynia

Our results indicate that the expression of CD11b was weaker than Iba1 (Figure 7 and Figure 8), which led to the subsequent dominance of Iba1+/CD11b− microglial cells in alcoholics in both gray and white matter in SNpr and SNpc (up to 40–50% of all microglial/macrophage population). Previous studies in mice-brain-derived microglial cells have demonstrated that CD11b silences TLR4 (toll-like receptor 4) -induced inflammatory responses [89]. Such physiological silencing in controls (wild-type mice) leads to increased microglial production of IL-10 and TGF-β (anti-inflammatory cytokines), coupled with decreased production of IL-6 and TNF-α (pro-inflammatory cytokines) [89], thereby indicating that silencing of CD11b promotes a pro-inflammatory microenvironment. Further, deficiency of CD11b has been shown to promote lipopolysaccharide-induced reactive oxygen species production, leading to the mice being more susceptible to endotoxin shock [86,90].

Recent studies have shown the involvement of SNpc and SNpr in the nociceptive pathways. SNpc and the ventral tegmental area receives nociceptive-related afferents from the parabrachial nucleus (PBN) and transfers these signals to subthalamic nucleus (STN) [91]. From STN, efferent signals reach SNpr which, in turn, sends signals to superior colliculus and PBN [92,93], thereby creating an anatomic nociceptive signal processing circuit. Alcohol has been implicated in modulating the reward mechanisms in STN (lesions in STN have been shown to decrease the motivation for alcohol intake) [94,95]. Such elevation of activity in STN by alcohol could lead to chronic pain and associated symptoms, as seen in Parkinson’s disease [91,96] and other neurodegenerative disease such as AD, motor neuron disease, Huntington’s disease, spinocerebellar ataxia, and spinal muscular atrophy [96,97]. Apart from the direct effects of alcohol on STN to increase pain sensitivity, we speculate that the dominance of CD11b− microglia in SN region also plays an amplifying role. CD11b−microglia-deficient mice models have been shown to be more susceptible to thermal and mechanical allodynia, which, in turn, have been attributed to increased microglial inflammatory response [89]. Altogether, our results indicate that attenuation of microglial CD11b in SN due to alcohol exposure leads to chronic and sustained inflammation, which leads to paradoxical withdrawal hyperalgesia and allodynia (via STN circuit), complaints commonly reported as part of alcohol withdrawal process [98,99].

### 4.5. Limitations of the Present Study

Nonetheless, the results obtained in the present study raise more questions than answers and are constrained by some limitations. Firstly, the number of investigated individuals per group was relatively low, and investigations in larger cohorts are needed. Secondly, to obtain a comprehensive view of the neuroinflammatory and degenerative changes seen in SN, a wider spectrum of immune markers is needed. Thirdly, we only investigated the role of microglia in alcohol and HHV-6 infection co-exposure-mediated changes in SN, and since other regulatory cells like astrocytes, macrophages, monocytes, neutrophils, etc. also take part in mediating these changes, future studies are needed to elucidate the specific roles of these cells. Finally, since HHV-6 infection precedes alcohol exposure (99% of us contract HHV-6 by the age of 2–3 years), it is difficult to elucidate whether HHV-6 infection amplifies alcohol-mediated damage or the other way around. Studies in animal models and cell cultures may shed some light on this matter in the future.

## 5. Conclusions

In the present study, we showed that the neuroinflammation-associated microglia changes in chronic alcoholics synergizes with the neuroinflammatory changes associated with HHV-6 infection, which results in a new, enhanced neuroinflammatory phenotype when both conditions are present. Further, the largely coherent agreement between the data from non-age matched alcoholics and age matched alcoholics enabled us to exclude ageing as an underlying covariate in the analysis. Finally, the following three conclusions are noteworthy.

Firstly, CD68+/Iba1− is the predominant microglial subpopulation in physiological conditions in the SN region and represents a “*controlled phagocytotic activated state*” with less migration-related and more phagocytotic activity. Alcoholics showed a significant decline of this subpopulation in both SNpc and SNpr.

Secondly, alcohol- and virus-induced immune exhaustion leads to progressive dystrophic degeneration of microglia via upregulation of Iba1 expression, leading to the emergence of CD68+/Iba1+ microglia (seen with less branching and beaded processes), which may be associated with neuroinflammatory changes. Microglia then compensate for these changes by increasing their migratory activities. Such a phenomenon is universal in all the regions of SN (SNpc and SNpr).

Finally, an indiscriminate increase in Iba1 expression when compared to CD11b in microglial cells in response to HHV-6 and alcohol exposure leads to the emergence of various subpopulations with Iba1+/CD11b− microglia being associated with the sustaining and promotion of neuroinflammatory changes in the SN region.

## Figures and Tables

**Table 1:** Grouping of individuals along with their age and gender.

Group A (Control Group)			Group B (Age-Matched Alcoholics)			Group C (Non-Age-Matched Alcoholics)		
Individual Code	Age (Years)	Gender (Male/ Female)	Individual Code	Age (Years)	Gender (Male/ Female)	Individual Code	Age (Years)	Gender (Male/ Female)
A1	34	M	B1	36	F	C1	48	M
A2	31	M	B2	23	M	C2	55	M
A3	27	M	B3	31	M	C3	60	M
A4	23	M	B4	26	M	C4	50	F
A5	32	M	B5	33	M	C5	63	M
A6	37	M	B6	34	F	C6	45	M
A7	33	M	B7	25	F	C7	45	M
A8	22	M	B8	30	M	C8	55	M
A9	36	M	B9	35	M	C9	45	F
A10	17	M	B10	22	M	C10	60	F
A11	37	M	B11	35	M	C11	66	M
A12	20	M	B12	34	M	C12	63	M
A13	26	M	B13	29	M	C13	44	F
						C14	49	M
						C15	45	F
						C16	60	M
						C17	38	F
						C18	40	M
Group median age		31 ± 6.79	Group median age		31 ± 4.85	Group median age		49.5 ± 8.66

**Table 2:** Description and characteristics of the primary antibodies.

Primary Antibody *	Antibody Characteristics **	Clone	Dilution	Manufacturer
CD68	Monoclonal mouse AB against human AG	Kp-1	1:200	Cell Marque (USA)
CD11b	Monoclonal rabbit AB against human AG	EP45	1:100	Epitomics (USA)
Iba1	Monoclonal rabbit AB against human AG	EP289	1:150	Epitomics (USA)
HHV-6 (20)	Monoclonal mouse AB against viral lysate	-	1:200	Santa Cruz (USA)

* CD68—cluster of differentiation 68; CD11b—cluster of differentiation 11b; Iba1—ionizing calcium-binding adaptor molecule 1; and HHV-6—human herpesvirus-6. ** AB, antibody; AG, antigen.

**Table 3:** Primers used for two-step PCR reaction for detection of HHV-6 variants.

Primer	Primer Sequence
Outer primer O1	5′-AGTCATCACGATCGGCGTGCTATC-3′
Outer primer O2	5′-TATCTAGCGCAATCGCTATGTCG-3′
Inner primer I3	5′-TCGACTCTCACCCTACTGAACGAG-3′
Inner primer I4	5′-TGACTAGAGAGCGACAAATTGGAG-3′

**Table 4:** Distribution of CD68+ cells per visual field in different regions of *Substantia Nigra* (SN).

Region	Group A	Group B	Group C	*p* Value ^†^
*Pars Compacta* (SNpc)				
Gray matter	6.50 ± 0.27	6.32 ± 0.20	6.22 ± 0.15	0.908
White matter	6.55 ± 0.25	6.07 ± 0.18	5.97 ± 0.20	0.360
*p* value ^‡^	0.749	0.145	0.519	-
*Pars Reticulata* (SNpr)				
Gray matter	6.50 ± 0.25	6.34 ± 0.22	5.61 ± 0.25	0.031 **
White matter	7.40 ± 0.29	6.75 ± 0.25	5.86 ± 0.19	<0.001 **
*p* value ^‡^	0.001 **	0.002 **	0.020 **	-

^†^*p* value was calculated for Kruskal-Wallis ANOVA (intergroup analysis). ^‡^ *p* value was calculated for related-samples Wilcoxon Signed Rank Test (intragroup analysis). The numbers represent the average number of CD68+ cells per visual field ± S.E. (standard error). ** indicates a significant difference between groups (*p* < 0.05 is considered significant with Bonferroni correction for Kruskal-Wallis ANOVA and without correction for related-samples Wilcoxon Signed Rank Test).

**Table 5:** Distribution of Iba1+ cells per visual field in different regions of *Substantia Nigra* (SN).

Region	Group A	Group B	Group C	*p* Value ^†^
*Pars Compacta* (SNpc)				
Gray matter	2.97 ± 0.15	4.41 ± 0.16	4.72 ± 0.17	<0.001 **
White matter	3.50 ± 0.17	4.57 ± 0.14	4.80 ± 0.17	<0.001 **
*p* value ^‡^	<0.001 **	<0.001 **	0.232	-
*Pars Reticulata* (SNpr)				
Gray matter	1.97 ± 0.13	2.20 ± 0.15	3.27 ± 0.14	<0.001 **
White matter	2.30 ± 0.16	2.45 ± 0.16	3.31 ± 0.13	<0.001 **
*p* value ^‡^	<0.001 **	0.342	0.752	-

^†^*p* value was calculated for Kruskal-Wallis ANOVA (intergroup analysis). ^‡^ *p* value was calculated for related-samples Wilcoxon Signed Rank Test (intragroup analysis). The numbers represent the average number of Iba1+ cells per visual field ± S.E. (standard error). ** indicates a significant difference between groups (*p* < 0.05 is considered significant with Bonferroni correction for Kruskal-Wallis ANOVA and without correction for related-samples Wilcoxon Signed Rank Test).

**Table 6:** Distribution of CD11b+ cells per visual field in different regions of *Substantia Nigra* (SN).

Region	Group A	Group B	Group C	*p* Value ^†^
*Pars Compacta* (SNpc)				
Gray matter	1.02 ± 0.07	1.47 ± 0.07	1.58 ± 0.09	<0.001 **
White matter	1.34 ± 0.09	1.60 ± 0.07	1.73 ± 0.09	0.005 **
*p* value ^‡^	0.002 **	0.191	0.165	-
*Pars Reticulata* (SNpr)				
Gray matter	0.72 ± 0.08	0.83 ± 0.08	0.88 ± 0.07	0.143
White matter	0.79 ± 0.09	0.83 ± 0.07	0.95 ± 0.09	0.724
*p* value ^‡^	0.815	0.270	0.603	-

^†^*p* value was calculated for Kruskal-Wallis ANOVA (intergroup analysis). ^‡^ *p* value was calculated for related-samples Wilcoxon Signed Rank Test (intragroup analysis). The numbers represent the average number of CD11b+ cells per visual field ± S.E. (standard error). ** indicates a significant difference between groups (*p* < 0.05 is considered significant with Bonferroni correction for Kruskal-Wallis ANOVA and without correction for related-samples Wilcoxon Signed Rank Test).

## Data Availability

All the data used in this study are available from the corresponding author upon request.
